# The Role of the TGF-β Superfamily in Myocardial Infarction

**DOI:** 10.3389/fcvm.2019.00140

**Published:** 2019-09-18

**Authors:** Anis Hanna, Nikolaos G. Frangogiannis

**Affiliations:** Department of Medicine (Cardiology), The Wilf Family Cardiovascular Research Institute, Albert Einstein College of Medicine, Bronx, NY, United States

**Keywords:** TGF-β, myocardial infarction, Smad, BMP, GDF, fibrosis, inflammation

## Abstract

The members of the transforming growth factor β (TGF-β) superfamily are essential regulators of cell differentiation, phenotype and function, and have been implicated in the pathogenesis of many diseases. Myocardial infarction is associated with induction of several members of the superfamily, including TGF-β1, TGF-β2, TGF-β3, bone morphogenetic protein (BMP)-2, BMP-4, BMP-10, growth differentiation factor (GDF)-8, GDF-11 and activin A. This manuscript reviews our current knowledge on the patterns and mechanisms of regulation and activation of TGF-β superfamily members in the infarcted heart, and discusses their cellular actions and downstream signaling mechanisms. In the infarcted heart, TGF-β isoforms modulate cardiomyocyte survival and hypertrophic responses, critically regulate immune cell function, activate fibroblasts, and stimulate a matrix-preserving program. BMP subfamily members have been suggested to exert both pro- and anti-inflammatory actions and may regulate fibrosis. Members of the GDF subfamily may also modulate survival and hypertrophy of cardiomyocytes and regulate inflammation. Important actions of TGF-β superfamily members may be mediated through activation of Smad-dependent or non-Smad pathways. The critical role of TGF-β signaling cascades in cardiac repair, remodeling, fibrosis, and regeneration may suggest attractive therapeutic targets for myocardial infarction patients. However, the pleiotropic, cell-specific, and context-dependent actions of TGF-β superfamily members pose major challenges in therapeutic translation.

## Introduction

In human patients, myocardial infarction is typically caused by thrombotic occlusion of a coronary artery, leading to prolonged cessation of blood flow in the territory subserved by the vessel. Ischemia-induced disruption of aerobic metabolism in cardiomyocytes immediately perturbs their contractile function and ultimately results in catastrophic structural changes (1). After 20–30 min of ischemia, subendocardial cardiomyocytes in the area at risk exhibit irreversible changes. Longer ischemic intervals trigger a “wavefront” of cardiomyocyte death that ultimately involves the entire thickness of the ventricular wall supplied by the occluded coronary (2). Because the adult mammalian heart has negligible regenerative capacity, repair of the infarcted myocardium is dependent on formation of a collagen-based scar, and requires activation of a superbly orchestrated inflammatory/reparative response (3). Inflammatory chemokines and cytokines are critically involved in the reparative process, mediating activation of macrophages that clear the wound from dead cells and matrix debris, and triggering expansion of reparative cells (including fibroblasts and vascular cells) that form granulation tissue and secrete a collagen-based matrix network. Although scar formation protects the heart from catastrophic early complications (such as cardiac rupture), infarct healing is closely intertwined with adverse remodeling, a constellation of cellular events that involve both infarcted and non-infarcted myocardium, and lead to chamber dilation, exacerbating dysfunction.

The members of the TGF-β superfamily play important roles in cell survival, differentiation, proliferation, and function and have been implicated in regulation of inflammatory and reparative responses. Following myocardial infarction, induction, and activation of endogenous TGF-β signaling pathways has been suggested to modulate injury, regulate inflammation, and orchestrate repair (4). This review manuscript discusses our current knowledge on the role of TGF-β superfamily members in injury, repair, and remodeling of the infarcted heart.

## The Members of the TGF-β Superfamily

In humans, the TGF-β superfamily is comprised of 33 members that can be subclassified into several subfamilies, including the TGF-βs (TGF-β1, -β2, and -β3), the bone morphogenetic proteins (BMPs), the Growth differentiation factors (GDFs), the activins, the inhibins, nodal, and anti-Mullerian hormone proteins. Although TGF-β1 remains the best-studied member of the superfamily in myocardial infarction, a growing body of evidence implicates several other members in regulation of cardiac repair and post-infarction remodeling.

## The TGF-βs

### TGF-β Expression in Normal Myocardium

High constitutive expression of TGF-β is noted in both embryonic and adult mammalian hearts, and is predominantly localized in cardiomyocytes, or bound to the extracellular matrix (5). TGF-βs play an important role in cardiac development; loss of TGF-β isoforms in neonatal mice is associated with abnormal cardiac development and defective valve formation (6, 7). In normal adult mammalian hearts, TGF-β is stored in a latent form; whether low-level constitutive TGF-β activity is required to support homeostatic cardiac function remains unknown. *In vivo*, targeting Smad4 (the common intracellular effector of responses triggered by many members of the TGF-β superfamily) in adult cardiomyocytes was associated with impaired ventricular function and with perturbations in ion channel gene expression (8). Moreover, in neonatal rat cardiomyocytes cultured on fibroblast matrix under serum-free conditions, exogenous TGF-β sustains spontaneous rhythmic beating (9). However, the *in vivo* relevance of this observation is unclear.

### Induction and Cellular Origin of TGF-βs in the Infarcted Myocardium

Upregulation of TGF-βs is well-documented in both mouse and large animal models of myocardial infarction (5, 10–15). In a mouse model of reperfused myocardial infarction, TGF-β1 and TGF-β2 mRNA levels peak early after 6–72 h of reperfusion; in contrast, TGF-β3 exhibits a prolonged time course and is persistently upregulated after 7 days of reperfusion (10). The distinct time course of TGF-β3 may be due to cell-specific expression patterns, or may reflect isoform-specific effects of stimuli inducing transcription of TGF-βs.

Although most myocardial cell types are capable of synthesizing and releasing significant amounts of TGF-βs; their relative contribution remains poorly defined. In a porcine model of coronary occlusion, cardiomyocytes were a major source of TGF-β (11). On the other hand, studies in mouse models of myocardial infarction suggest that infarct macrophages may be key contributors to the TGF-β response (16). Genetic disruption of the chemokine monocyte chemoattractant protein (MCP)-1/CCL2, a mediator with a crucial role in recruitment of monocytes/macrophages in inflamed tissues, was associated with reduced TGF-β2 and TGF-β3 mRNA expression following myocardial infarction, consistent with an important role for infiltrating mononuclear cells in TGF-β synthesis in the infarcted heart (17). TGF-β synthesis may mark specific subsets of infarct macrophages that respond to cytokine stimulation in the pro-inflammatory environment of the infarct (18). In addition to cardiomyocytes and macrophages, several other cell types may also secrete TGF-βs in the infarcted heart; however, robust documentation of their contribution is lacking. Platelets constitutively express significant amounts of growth factors and have been suggested to be an important source of TGF-β1 in the pressure-overloaded myocardium (19). Abundant platelets infiltrate the infarct during the early stages of repair; however, their relative role as a source of TGF-βs has not been investigated. Activated fibroblasts, vascular cells, mast cells and lymphocyte subsets can produce and secrete TGF-βs, and are found in significant numbers in infarcted hearts (20–23). However, whether they are a significant source of TGF-β isoforms following infarction remains unknown.

### TGF-β Activation in the Infarcted Myocardium

In the infarcted myocardium, activated cells enrich the existing stores of latent TGF-β through *de novo* synthesis of all 3 isoforms. However, this is not sufficient for activation of TGF-β cascades. Transduction of TGF-β signals in sites of injury requires liberation of the active TGF-β dimer from the latent complexes. TGF-βs are secreted in a latent form that is comprised of the TGF-β dimer, the latency-associated peptide (LAP), which confers latency to TGF-β (24, 25), and a latent TGF-β-binding protein (LTBP), which serves to sequester the complex into the extracellular matrix (26). Extensive data from animal models suggest that myocardial infarction is associated with rapid activation of TGF-β (27), followed by stimulation of downstream Smad-dependent signaling cascades (28). However, the molecular signals that trigger TGF-β activation following infarction remain poorly understood. Several mechanisms may be involved. First, cell surface integrins may interact with the LAP, thus releasing the TGF-β dimer (29). Although *in vitro* studies have implicated αvβ5 and αvβ3 integrins in latent TGF-β activation and in subsequent myofibroblast conversion (30); the *in vivo* significance of these interactions in the infarcted myocardium remains unknown. Second, a wide range of proteases (including serine proteases, cathepsins, matrix metalloproteinases, and cysteine proteases) have been implicated in TGF-β activation following injury. Proteases are rapidly activated following myocardial infarction (31–33); however, their potential role in activation of TGF-β in infarcted hearts remains poorly documented. Third, reactive oxygen species (ROS) are rapidly generated in ischemic hearts and may be involved in activation of TGF-β activation in the infarcted myocardium. Fourth, specialized extracellular matrix proteins (including matricellular proteins, such as thrombospondin-1), are markedly upregulated in the infarcted heart (34–36) and may play an important role in TGF-β activation. Thrombospondin-1 has been suggested to interact with the LAP, promoting release of the active TGF-β dimer from the latent complex (37). In the infarcted myocardium, TSP-1 upregulation is associated with activation of TGF-β signaling in the infarct border zone in both mouse and canine infarcts (34). Finally, an acidic environment has been suggested to promote TGF-β activation (38). Generation of lactic acid in the ischemic myocardium may reduce the pH, contributing to activation of latent TGF-β stores following infarction.

### Cellular Actions of TGF-β in the Infarcted Myocardium (Figure 1)

TGF-β1, β2, and β3 are activated in the infarcted myocardium and may regulate phenotype and function of all cell types involved in cardiac injury and repair. Although the 3 TGF-β isoforms exhibit distinct patterns of regulation following infarction, little is known regarding isoform-specific actions in the infarcted heart. Most *in vitro* studies have investigated TGF-β1-mediated actions. *In vivo* experiments on the other hand, have focused on the role of TGF-β receptor-activated signaling, exploring pathways common to all three isoforms and to other members of the TGF-β superfamily.

**Figure 1:**
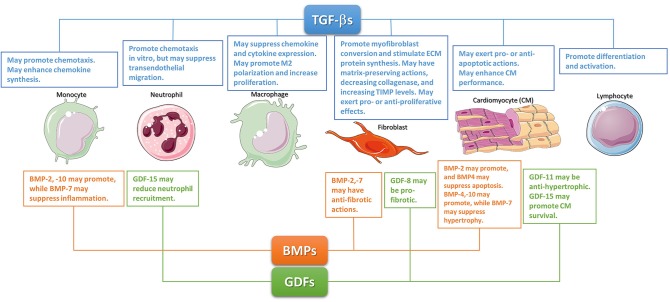
The diverse cellular actions of the TGF-βs, BMPs, and GDFs in the infarcted myocardium. During the inflammatory phase of infarct healing, TGF-βs may regulate cardiomyocyte survival, promote monocyte chemotaxis, and modulate lymphocyte differentiation and activation. TGF-βs may be critical in modulating macrophage phenotype toward an anti-inflammatory M2 phenotype. These effects may act as a switch from inflammation to repair. The effects of BMPs and GDFs have not been systematically studied; however, several members of these subfamilies may regulate inflammation. During the reparative phase, TGF-βs are critical regulators of fibroblast activation. Although studies in cardiac fibroblasts suggested TGF-βs can have either pro- or anti- proliferative effects, TGF-βs have consistent effects on fibroblast activation, mediating myofibroblast conversion and stimulating exctracellular matrix protein synthesis. Moreover, TGF-βs also promote a matrix-preserving program by decreasing collagenase synthesis, and by upregulating tissue inhibitors of metalloproteinases (TIMPs). Members of the GDF and BMP subfamilies may also regulate phenotype and function of reparative cells. The cartoon was designed using Servier Medical Art (https://smart.servier.com/).

#### Effects of TGF-β on Ischemic Cardiomyocytes

Early studies in isolated perfused hearts undergoing brief myocardial ischemia followed by reperfusion, suggested protective effects of TGF-β, mediated through attenuation of oxidative stress and reduced release of pro-inflammatory cytokines, such as tumor necrosis factor (TNF)-α (39). *Ex vivo* and *in vitro* experiments examining the effects of TGF-β on cardiomyocyte survival have produced conflicting results. In a model of reperfused feline myocardial infarction, TGF-β1 administration reduced cardiomyocyte death, and attenuated neutrophil recruitment in the infarcted myocardium (40). It is unclear whether TGF-β-mediated protection in these studies was due to activation of direct pro-survival effects in cardiomyocytes, or reflected suppression of injurious inflammatory signaling. TGF-β1 infusion in isolated perfused hearts, protected cardiomyocytes from apoptosis through actions involving p42/p44 mitogen-activated protein kinase (MAPK) signaling during early reperfusion (41). In contrast, in rat cardiomyocytes, the pro-apoptotic effects of angiotensin II were attributed to activation of TGF-β signaling (42). Moreover, *in vitro* studies have suggested direct actions of TGF-β on cardiomyocyte function, mediated through upregulation of the laminin receptor 37/67 (43). Considering its notoriously pleiotropic and context-dependent actions, the effects of exogenous TGF-β administration are likely dose- and time-dependent, and are affected by the experimental conditions.

*In vivo*, mouse models of cardiomyocyte-specific TGF–β receptor disruption have been used to investigate endogenous effects of TGF–βs on cardiomyocytes (44), In a model of non-reperfused myocardial infarction, mice with cardiomyocyte-specific loss of the type 1 or type 2 TGF-β receptor were protected from death due to cardiac rupture, exhibiting marked reduction in neutrophil infiltration. The mechanism of protection remains poorly defined. It was suggested that TGF-β signaling in cardiomyocytes may suppress synthesis of cardioprotective genes, such as interleukin (IL)-33, GDF-15, and TSP-4.

#### TGF-β and the Post-Infarction Inflammatory Reaction

TGF-βs are critical modulators of immune cell phenotype and function (45). *In vitro*, TGF-βs can exert pro- or anti-inflammatory depending on the phenotype and the state of differentiation of the cells, and the presence or absence of other mediators. TGF-β is a potent neutrophil (46) and monocyte (47) chemoattractant; these effects may be important for recruitment of leukocytes in inflamed tissues. Despite exhibiting strong chemotactic actions for monocytes and neutrophils in single-cell assays, TGF-β may attenuate transendothelial leukocyte migration by reducing surface expression of adhesion molecules (48). Moreover, in monocytes picomolar concentrations of TGF-β stimulate synthesis of pro-inflammatory cytokines and chemokines (47, 49). In contrast to its pro-inflammatory actions in monocytes, TGF-β is known to deactivate macrophages, suppressing expression of chemokines (such as MCP-1/CCL2) and cytokines, (including IL-1β and TNF-α) (50–52). Whether TGF–β acts as a pro- or anti-inflammatory mediator *in vivo* is dependent on the context: the cell types involved, their phenotypic profile, and the cytokine environment. Thus, attribution of pro- or anti-inflammatory properties to TGF-βs should be based on documentation of robust *in vivo* effects, rather than on *in vitro* assays.

*In vivo*, TGF-β1 plays an essential role in immune cell homeostasis by preventing spontaneous inflammation in mammalian tissues. 50% of TGF-β1 null mice develop massive multi-organ inflammation 2–4 weeks after birth, mainly affecting the heart and lungs (53). The anti-inflammatory effects of TGF-β in normal tissues may involve suppressive actions on T cell inflammatory activity (54).

In the healing infarct, timely recruitment of inflammatory leukocytes, and subsequent activation of reparative macrophages play a crucial role in repair, but may also mediate fibrotic responses that promote adverse remodeling (3, 55, 56). TGF–β may critically regulate inflammatory leukocyte recruitment and function following myocardial infarction. *Ex vivo* studies using cardiac lymph collected following reperfused infarction as a window to the cardiac microenvironment suggested that TGF-β activity may be implicated in recruitment of inflammatory monocytes in the healing infarct (27). *In vivo* experiments have suggested anti-inflammatory actions of TGF-β following infarction. Early systemic inhibition of TGF-β signaling through transfection with the extracellular domain of TβRII increased mortality in a model of non-reperfused infarction, accentuating neutrophil recruitment, and inducing expression of cytokines and chemokines (57). The specific effects of TGF-β signaling on the phenotype of immune cell subpopulations infiltrating the infarcted myocardium have not been systematically studied. TGF-β may be a critical modulator of macrophage phenotype, promoting M2 polarization (58) and suppressing expression of inflammatory cytokines and chemokines (58, 59), enhancing macrophage-colony stimulating factor (M-CSF)-induced proliferation (60), inhibiting nitrite release (61), and reducing cytotoxic activity (62). TGF-β also acts as a central mediator in T lymphocyte differentiation and activation, critically regulating phenotype and function of all subpopulations (63–65). CD4+ T cells (66) and foxp3+ regulatory T cells (Tregs) (20–22), have been implicated in regulation of repair and remodeling following myocardial infarction Whether TGF-β signaling cascades play a crucial role in differentiation and phenotypic modulation of lymphocytes in the infarcted heart remains unknown.

#### Effects of TGF-β on Fibroblast Phenotype and Function

During the reparative phase of cardiac repair, resident cardiac fibroblast populations become activated (67, 68), undergoing myofibroblast conversion, and secreting large amounts of structural extracellular matrix proteins (69–73). Activated infarct fibroblasts also produce cytokines, growth factors, and matricellular proteins, and participate in matrix metabolism through secretion of MMPs. TGF-β plays a crucial role in expansion of the myofibroblast population in the healing infarct and stimulates expression of extracellular matrix proteins. TGF-β potently induces expression of α-smooth muscle actin in cardiac fibroblasts, the hallmark of myofibroblast conversion (74, 75). Moreover, TGF-β significantly and consistently stimulates synthesis and secretion of extracellular matrix proteins (such as collagen I, collagen III, and fibronectin) (76, 77), and promotes a matrix-preserving phenotype by suppressing collagenase expression, and by accentuating synthesis of tissue inhibitor of metalloproteinases (TIMPs) (28, 78, 79). Whether TGF-β stimulates fibroblast proliferation in the infarcted heart is less clear. *In vitro* studies have demonstrated both proliferative and anti-proliferative effects of TGF-β on cardiac fibroblasts (77, 80). Differences in TGF–β concentration, distinct phenotypic profiles, or states of differentiation of fibroblast populations, and differences in the growth factor and extracellular matrix environment may account for the conflicting observations.

Although genetic interventions to study the *in vivo* effects of fibroblast-specific TGF-β signaling in infarcted hearts have not been performed, a large body of evidence suggests that TGF-β may play a critical role in regulation of fibroblast phenotype in healing infarcts. In a model of non-reperfused infarction, TGF-β inhibition through administration of a neutralizing antibody had detrimental effects, accentuating chamber dilation, increasing myocardial MMP expression, and reducing collagen synthesis (81). Moreover, two independent investigations inhibiting TGF-β through gene transfer of the extracellular domain of TβRII suggested that TGF-β may play a crucial role in fibrosis of the infarcted heart (57, 82). TGF-β inhibition after the inflammatory phase of cardiac repair attenuated deposition of fibrous tissue in the infarcted region (82). The observed effects may reflect, at least in part, loss of TGF-β signaling in cardiac fibroblasts.

#### Effects of TGF-β on Endothelial Cell Phenotype and on Infarct Angiogenesis

Endothelial cells, the most abundant non-cardiomyocytes in the adult mammalian myocardium (83), play an important role in regulation of the post-infarction inflammatory response and in repair of the infarcted heart. During the inflammatory phase of infarct healing, endothelial cells undergo activation, producing large amounts of chemokines (84, 85), and expressing adhesion molecules. Stimulated endothelial cells interact with activated circulating leukocytes promoting their recruitment in the healing infarct (86). During the proliferative phase, angiogenic activation of endothelial cells (87) may be important for generation of neovessels that supply the healing tissue with oxygen and nutrients. Finally, during the maturation phase, endothelial cells may recruit mural cells, by producing platelet-derived growth factor-BB (88, 89); thus restraining angiogenesis, suppressing inflammation and contributing to formation of a mature scar (90).

Although both endothelial cells and pericytes are highly responsive to TGF-β (91), the role of TGF-β-mediated actions on the infarct microvasculature remains unknown. It has been suggested that suppressive effects of TGF-β on endothelial cell chemokine synthesis may contribute to suppression and resolution of the post-infarction inflammatory response (92). TGF-β may also be implicated in regulation of infarct angiogenesis and may contribute to vascular maturation by modulating interactions between endothelial cells and mural cells. It should be emphasized that TGF-β actions on endothelial cells may be either angiogenic, or angiostatic, depending on contextual factors (91, 93).

## TGF-β and Cardiac Regeneration

The adult mammalian myocardium has extremely limited regenerative capacity that is overwhelmed by the massive loss of cardiomyocytes following myocardial infarction. Accentuation of the regenerative capacity of the heart in order to remuscularize the infarcted myocardium is a major visionary goal in cardiovascular research. Experimental evidence suggests a broad and context-dependent role for TGF-β in cardiac regeneration. TGF-β may regulate cardiomyocyte differentiation *in vitro* (94), may be involved in endogenous remuscularization in regenerative models of cardiac injury (95), and may modulate regenerative responses to cell therapy following myocardial infarction (96). An *in vitro* study showed that TGF-β stimulation increases expression of cardiac transcription factors in murine embryonic stem cells driving cardiomyocyte differentiation (97). In contrast, in other *in vitro* experiments, a highly selective TGF-β inhibitor that promotes proteasomal degradation of TβRII enhanced differentiation of uncommitted mesoderm to cardiomyocytes (98). *In vivo* studies in fish models have supported the involvement of TGF-β in regulation of endogenous cardiac regeneration. In zebrafish, pharmacologic inhibition of TβRI abolished heart regeneration following cryoinjury (95). In contrast, a pharmacological inhibitor of the type I TGFβ receptor was found to enhance proliferation of Nkx2.5+ cardiomyoblasts (99), and improved endogenous cardiomyoblast-mediated regeneration in mice (96). TGF-β has also been found to modulate the effects of cell therapy. In a mouse model of myocardial infarction, intramyocardial implantation of TGF-β pre-programmed CD117+ stem cells was reported to promote regeneration and stimulate angiogenesis (100). The conflicting findings from various studies likely reflect the differences between experimental models and the context-dependent actions of TGF-β that typically depend on the state of differentiation of target cells. Considering that high levels of active TGF-β in the infarcted mammalian myocardium are not associated with any significant regenerative activity, it is highly unlikely that stimulation of the TGF-β cascade may be sufficient to promote remuscularization following cardiac injury. However, through its broad and pronounced effects on differentiation of many cell types, TGF-β may modulate phenotype and function of myocardial progenitor cells, thus critically regulating their regenerative capacity.

## The BMP/GDF Subfamily

### BMPs

BMPs are a group of evolutionarily conserved, structurally related secreted cytokines with an essential role in development and in the pathogenesis of many pathologic conditions (101). Although the name reflects the well-documented osteo-inductive properties of many members of the group, BMPs are pleiotropic and multifunctional, exerting a wide range of actions in many different cell types (102). Development of genetic loss of function models has documented the role of several BMPs in cardiac development (103, 104). Unfortunately, the potential involvement of BMPs in repair, remodeling and fibrosis of the infarcted heart remains poorly investigated.

#### BMP2

*In vitro*, BMP2 induces differentiation of cardiac progenitor cells into mature cardiomyocytes, stimulating expression of cardiac-specific genes (105). Knockout models in mice have demonstrated that BMP2 is critically involved in cardiac development (106), coordinating atrioventricular canal morphogenesis (107). Whether BMP2 plays a role in homeostatic function of the adult heart remains unclear. *In vitro*, BMP2 was found to stimulate contraction in adult rat cardiomyocytes, through activation of PI-3K (108).

Following myocardial infarction, BMP2 shows an early time course of upregulation. In a model of non-reperfused myocardial infarction, transient early induction of BMP2 was followed by increased expression of other BMPs (109). BMP2 expression in infarcted hearts was localized in both cardiomyocytes and interstitial fibroblasts (110, 111); however, the mechanism of upregulation remains unknown. Very little is known regarding the function of endogenous BMP-2 in myocardial infarction. Accentuation of post-infarction inflammation in mice lacking the BMP inhibitor gremlin (109) may suggest important pro-inflammatory actions of BMPs. Whether neutrophils, macrophages and lymphocytes, essential effector cells of post-infarction inflammation, are major targets of BMP2 is unknown. Moreover, BMP2 may exert cytoprotective actions on cardiomyocytes. *In vitro*, BMP2 inhibits apoptosis of serum-deprived cardiomyocytes (112). In mice undergoing permanent coronary occlusion protocols, intravenous BMP-2 injection attenuated apoptosis of infarct border zone cardiomyocytes, reducing the size of the infarct (113). Although inhibitory effects of BMP-2 on fibroblast function have been reported (114), the significance of such actions in repair following myocardial infarction and in cardiac fibrosis remains unclear. Angiogenic effects of BMP2 have been documented *in vitro* and *in vivo* (115); however, the potential role of BMP2 as a regulator of neovessel formation in healing myocardial infarction has not been investigated (Figure 1).

#### BMP4

Much like BMP2, BMP4 also promotes cardiomyocyte differentiation of cardiac progenitor cells (116, 117) Defective atrioventricular septation in BMP4 KO mice suggests a critical role for BMP4 in cardiac development (118). Adult human and mouse hearts exhibit constitutive BMP4 expression that increases following myocardial infarction (109, 119). The molecular mechanisms and predominant cellular sources responsible for BMP4 upregulation following infarction remain unknown. Limited information is available on the role of BMP4 following myocardial infarction. *In vitro* BMP4 has been suggested to promote apoptosis in neonatal rat cardiomyocytes (120). *In vivo*, in a model of ischemia/reperfusion, heterozygous BMP4 +/– null mice had reduced infarct size, and attenuated cardiomyocyte apoptosis (121). BMP4 may also stimulate a hypertrophic response in cardiomyocytes of the infarct border zone or the remote remodeling myocardial areas (122). In addition to effects on cardiomyocytes, BMP4 may also modulate inflammatory, fibrogenic and angiogenic responses. *In vitro* studies using endothelial cells and macrophages have suggested both pro- and anti-inflammatory actions of BMP4 (123, 124). BMP4 also stimulates angiogenic activation of endothelial cells through a Smad1/5-mediated pathway (125). Unfortunately, experimental studies exploring the role of these BMP4-mediated actions in myocardial infarction have not been performed (Figure 1).

#### BMP6

BMP6 is critically involved in iron homeostasis. Targeted disruption of BMP6 induces rapid and massive deposition of iron in many organs, including the heart (126). Iron overload in BMP6 null mice is associated with marked reduction of hepcidin (126), a key regulator of iron transport. BMP6 is expressed in developing hearts and mice with combined BMP6 and BMP7 loss exhibit a delay in formation of the outflow tract endocardial cushions (127). Although a clinical study showed increased circulating levels of BMP6 in patients with advanced heart failure (128), information on the regulation and role of BMP6 in the infarcted myocardium is lacking.

#### BMP7

Although BMP7 is highly expressed in the developing myocardium (129), BMP7 loss does not affect cardiac development, and defects in BMP7 null mice are confined to the kidney and the eye (130). A systematic study of myocardial BMP expression in non-reperfused myocardial infarcts found no significant upregulation of BMP7 mRNA expression 1–21 days following coronary occlusion, but suggested trends toward reduced levels at early timepoints (109). BMP7 exerts important actions on fibroblasts, cardiomyocytes and macrophages. Anti-fibrotic effects of BMP7 have been reported in many systems and may be mediated, at least in part, through suppression of collagen synthesis by cardiac fibroblasts (131), and through inhibition of endothelial to mesenchymal transition (EndMT) (132). In cardiomyocytes, BMP7 attenuates hypertrophy by inhibiting TGF-β responses (131). In macrophages, BMP7 has been reported to promote M2 polarization (133). Considering the absence of endogenous BMP7 induction in infarcted and remodeling hearts, administration of exogenous BMP7 has been suggested as a potential strategy to attenuate fibrosis and adverse remodeling. In a rat model of non-reperfused infarction systemic administration of BMP7 was reported to reduce mortality and attenuate ventricular dysfunction (134). Although the protective effects of exogenous BMP7 were attributed to anti-fibrotic actions; dissection of the cellular targets and molecular mechanisms was not performed.

#### BMP10

In the developing heart, BMP10 is regulated by myocardin and plays an important role in cardiomyocyte proliferation and chamber maturation (135, 136). In the postnatal heart, BMP10 expression seems to be restricted to the right atrium (137). Experiments in a non-reperfused mouse model of myocardial infarction showed a 2-fold increase in BMP10 levels 21 days after coronary occlusion. The cellular source of BMP10 in the infarcted heart, and the basis for the delayed pattern of induction remain unknown. Moreover, the effects of endogenous BMP10 in repair and remodeling of the infarcted heart have not been investigated. *In vitro* assays demonstrated that BMP10 enhances TNF-α-driven monocyte recruitment to the vascular endothelium promoting inflammation (138). However, considering the late time course of induction in the healing infarct, pro-inflammatory effects of BMP10 are unlikely to play an important role in the infarcted heart. BMP10 has been also reported to exert pro-hypertrophic actions in cardiomyocytes through an interaction with titin-cap (Tcap) (139). A study in a rat model of myocardial infarction suggested that exogenous BMP10 administration may stimulate cardiomyocyte cell cycle reentry (140). Considering the negligible proliferative activity of cardiomyocytes in infarcted and remodeling hearts, despite increased BMP10 levels and the challenges in robust documentation of proliferating cardiomyocytes, the significance of these findings in cardiac repair is unclear.

### GDFs

Growth/differentiation factors (GDF1 to 15) are produced as inactive precursor proteins, which are then cleaved and assembled into active secreted homodimers. GDF dimers are disulfide-linked ligands that bind to a diverse array of receptors. (141, 142).

#### GDF8

GDF8 (also known as myostatin) is a critical negative regulator of growth and mass of skeletal and cardiac muscle, and plays an important role in energy homeostasis in adult cardiomyocytes. Cardiomyocyte-specific deletion of GDF8 in adult mice is associated with cardiomyocyte hypertrophy and heart failure, by promoting AMP-activated kinase activation (141). Following infarction, rapid upregulation of GDF8 has been reported within minutes after coronary ischemia (143). Surviving cardiomyocytes in the border zone of the infarct have been suggested as the main cellular source of GDF8 in infarcted and remodeling hearts (142). The functional role and cellular targets of GDF8 in the infarcted heart remain poorly understood. A study using a global loss-of-function model GDF8 loss may protect the infarcted heart, attenuating late dysfunction. The protective effects of GDF8 loss were attributed to attenuated fibrosis (144). The findings of this study are difficult to interpret, considering the baseline effects of myostatin loss on the myocardium, and the challenges in identifying cell-specific mechanisms. Pro-fibrotic actions of GDF8 in the myocardium may involve direct activation of fibroblasts (145–147), or effects on other cell types such as cardiomyocytes and macrophages.

#### GDF11

Publication of a high impact study suggesting that GDF11 levels decline with aging and that GDF11 administration may reverse age-related hypertrophy (148) (Figure 1) generated great interest in the potential role of this approach as therapy to reverse aging in many tissues (149). Subsequent studies have challenged the notion that GDF11 can rejuvenate the aging heart (150). Thus, additional work is necessary to settle this controversy. Although effects of GDF11 on hypertrophic responses have been suggested in models of left ventricular pressure overload (151), the regulation, cellular effects and actions of endogenous GDF11 in the infarcted myocardium have not been systematically investigated. Exogenous delivery of GDF11 was found to be protective in mouse and rat models of myocardial ischemia/reperfusion. (152, 153) Beneficial effects of GDF11 were attributed to improved angiogenesis and homing of endothelial progenitors (152), or to attenuated oxidative stress (153).

#### GDF15

GDF-15 is an inflammation-associated hormone with an important protective role in a wide range of infectious and inflammatory conditions (154, 155). In the mammalian myocardium, GDF15 is secreted by cardiomyocytes and may regulate body growth. In pediatric patients with heart disease, increased secretion of GDF15 negatively regulates body growth through effects on liver-derived growth hormone signaling (156). GDF15 expression is rapidly increased following myocardial ischemia and is predominantly localized in cardiomyocytes (157). Extensive experimental evidence suggests a crucial role for GDF15 in regulation of cardiomyocyte survival, post-infarction inflammation, repair, and remodeling of the infarcted heart. In a model of reperfused myocardial infarction, GDF15 exerted pro-survival actions on cardiomyocytes, reducing the size of the infarct (157). Moreover, in a model of non-reperfused infarction, GDF15 protected from cardiac rupture, restraining neutrophil recruitment through suppression of leukocyte integrin activation (155). Clinical studies have suggested that serum GDF15 levels may improve risk stratification in both STEMI and non-STEMI patients, providing important prognostic information beyond established clinical and biochemical markers (158–160) (Figure 1).

## The Inhibin/Activin Subfamily and the Role of the Follistatins

Activins are dimeric polypeptides composed of two β subunits of inhibin linked by a disulfide bridge (161). Five types of β subunits have been described (βA, βB, βC, βD, and βE); activin nomenclature is defined by the type of subunits that form the dimer. In mammals, activins A (βA/βA), B (βB/βB), AB (βA/βB), C (βC/βC), and E (βE/βE) have been isolated; however, only activins A, B and AB have been studied and are known to exhibit biological activity (162).

In a rat model of non-reperfused myocardial infarction activin βA was markedly upregulated after 2 days of coronary occlusion and remained elevated for at least 28 days. Activin A protein was predominantly localized in surviving cardiomyocytes (163). A very rapid upregulation of activin A was reported in a model of reperfused myocardial infarction, occurring as early as 30 min after coronary ischemia. Early induction of activin A was dependent on TLR4 signaling (164). Overexpression experiments *in vivo* and *in vitro* suggested that activin A may protect ischemic cardiomyocytes from apoptosis (165). Activin A may also activate fibroblasts, promoting cell proliferation and collagen synthesis through p38 and Erk MAPK pathways (166).

The activin system is regulated by follistatin and follistatin-like 3 (Fstl3), extracellular proteins that bind activins with high affinity to form inactive complexes. In a rat model of myocardial infarction, administration of exogenous follistatin was reported to attenuate inflammation (167). Endogenous Flstl3 is upregulated following myocardial infarction and attenuates the cardioprotective effects of activin A (165). Cardiomyocyte-derived Flstl3 may also exert hypertrophic actions (168) and may stimulate fibroblasts in a paracrine manner, promoting a fibrogenic phenotype (169). It should be emphasized that Flstl3 actions *in vivo* may be, at least in part, independent of activin inhibition. Activin actions may also be relevant in human ischemic heart disease. Patients with both ischemic and non-ischemic heart failure had elevated circulating levels of activin A (163).

## Signaling Pathways Activated by the TGF-β Superfamily in the Infarcted Myocardium (Figure 2)

The members of the TGF-β superfamily signal through binding to dual specificity kinase receptors (that have characteristics of both serine/threonine and tyrosine kinases) on the cell surface. Binding of a TGF-β family member assembles a heterotetrameric complex of 2 type 1 and 2 type II TGF-β receptors (170, 171). In humans, there are 7 type I receptors, termed activin-like receptor kinase (ALK)1-7, and 5 type II receptors; each member of the TGF-β superfamily signals by binding to characteristic combinations of these receptors. TGF-β isoforms transduce signaling through a single type II receptor (TβRII); in contrast, most BMPs are more promiscuous binding to three different type II receptors (ActRII, ActRIIB, and BMPRII). Binding of TGF-βs superfamily members to the type II receptor promotes oligomerization and transphoshorylates a type I receptor kinase. Activation of specific type I receptors accounts for part of the cellular specificity of TGF-β actions. For example, in most cell types TGF-βs activate the ubiquitously expressed type I receptor ALK5 (172). In endothelial cells, TGF-βs exerts distinct effects through another type I TGF-β receptor, ALK1 (173). Subsequently, activated type I receptors phosphorylate intracellular transcriptional effectors, the receptor-activated Smads (R-Smads). TGF-β-induced ALK5 activation, or activin-mediated ALK4 stimulation typically trigger phosphorylation of Smad2 and Smad3. TGF-β-induced ALK1 activation or BMP signaling generally phosphorylate Smad1, Smad5, and Smad8. Activated R-Smads form complexes with the common Smad, Smad4, and translocate to the nucleus, where they recruit co-activators or co-repression of transcriptional complexes and modulate gene transcription (174). In addition to their effects mediated through activation of Smads, the members of the TGF-β superfamily also signal through non-Smad pathways, by activating mitogen-activated protein kinase (MAPK) family responses, PI3K/Akt, focal adhesion kinase (FAK), and Rho GTPase pathways (175, 176). The multiple interactions between signaling responses of TGF-β superfamily members and other cascades (such as Wnt and Notch signaling) (177) generate additional layers of complexity that may explain, at least in part, the context-dependent actions of TGF-βs.

**Figure 2:**
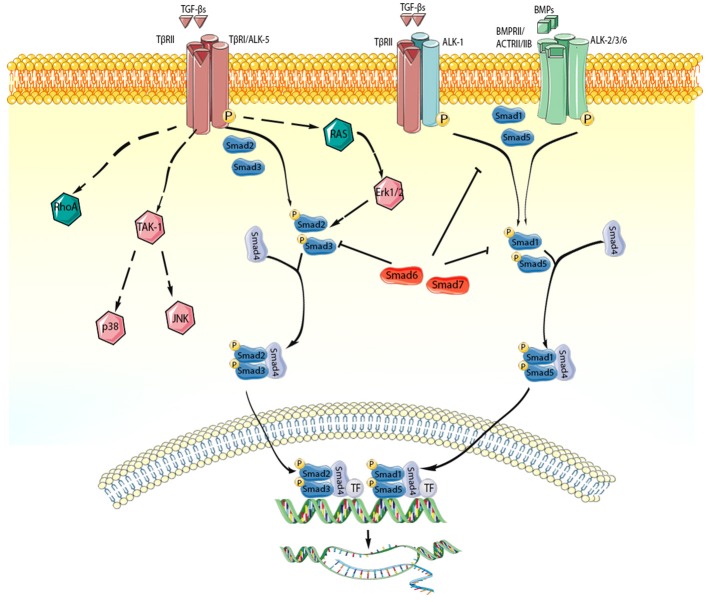
TGF-β superfamily members act through activation of Smad-dependent and Smad-independent signaling pathways. Each member of the TGF-β superfamily signals by binding to distinct combinations of type II, and type I receptors. TGF-β dimers bind TβRII inducing transphosphorylation of the GS segments in the type I receptor (ALK-5). Activated ALK-5 phosphorylates the receptor-activated Smads (R-Smads) Smad2 and Smad3, which then form a heterotrimeric complex with the common Smad, Smad4, promoting the translocation of the Smad complex to the nucleus. Interactions between the Smad and transcriptional activators or repressors regulate transcription of target genes. In some cell types (such as endothelial cells), TGF-βs may act through the type I receptor ALK1, stimulating Smad1/5/8 signaling. BMP dimers on the other hand bind their specific type II receptors, subsequently activating type I receptors (ALK-2,-3,-6) and phosphorylating Smad1/5. Smad1 and Smad5 then bind to Smad4 and translocate to the nucleus regulating transcription. In addition, TGF-β superfamily members signal through non-canonical Smad-independent cascades. The inhibitory Smads (Smad6 and Smad7) negatively regulate TGF-β superfamily cascades. The cartoon was designed using Servier Medical Art (https://smart.servier.com/).

### Smad-Dependent Signaling in Myocardial Infarction

#### The ALK5-Smad2/3 Cascade in the Infarcted Heart

Activation of Smad2 and Smad3 signaling is consistently noted in infarcted hearts, and is localized in border zone cardiomyocytes, fibroblasts, and immune cells (13, 28, 178–180). ALK5 inhibition attenuated Smad2 activation in healing infarcts (181). However, the relative contribution of various TGF-β superfamily members in activating Smad2 and Smad3 remains unclear. *In vivo* experiments using cell-specific knockout strategies suggested that Smad3 signaling exerts important actions in regulating cardiomyocyte, fibroblast, and macrophage function following myocardial infarction. In cardiomyocytes, Smad3 activation increased apoptosis, enhancing nitrosative stress, and increasing expression of MMPs in the remodeling myocardium (178). On the other hand, activation of Smad3 in myofibroblasts protected the infarcted heart from late rupture, and played a major role in repair of the infarct, promoting formation of organized arrays of reparative myofibroblasts in the border zone, through activation of an integrin-ROS axis (178). Macrophage-specific Smad3 activated a phagocytic program and played a major role in transition of macrophages to an anti-inflammatory phenotype (179). In contrast, Smad2 activation may have more subtle and limited effects in cardiac repair. Myofibroblast-specific loss of Smad2 was associated with a transient improvement in post-infarction remodeling, without affecting mortality, scar organization, or long-term outcome (180).

#### The Role of the Smad1/5/8 Cascade in Myocardial Infarction

Although the Smad1 cascade is also activated in the ischemic and infarcted heart (182–184) relatively little is known regarding its role in regulation of phenotype and function of surviving cardiomyocytes, immune cells, fibroblasts, and vascular cells. Whether Smad1 activation in infarcts is mediated predominantly through BMPs, or reflects effects of TGF-βs on cells expressing ALK1 is unknown. Cardiomyocyte-specific overexpression of Smad1was found to be protective, reducing infarct size in a model of ischemia/reperfusion, by attenuating activation of a pro-apoptotic program (182). However, the potential role of endogenous Smad1 activation in regulation of cardiac injury, repair, and remodeling has not been investigated.

### Activation of Smad-Independent Pathways in the Infarcted Heart

Although the members of the TGF-β superfamily are known to activate a wide range of non-canonical cascades, (including MAPK, TAK-1, and Rho GTPase) (177, 185, 186), the relative contribution of these pathways in myocardial infarction remains unknown. *In vitro* experiments and associative data have suggested that activation of a TAK-1/p38 MAPK axis by TGF-β may be involved in hypertrophic remodeling of surviving myocardium in the infarcted heart (187). In cardiac fibroblasts, stimulation of p38 MAPK may play an important role in fibrogenic activation (188) and may mediate hypertrophy (189). However, considering the broad range of mediators that signal through MAPKs, the role of TGF-β-driven activation of p38 in the infarcted heart is unclear.

## Targeting the TGF-β Superfamily in Myocardial Infarction

Considering the critical role of TGF-β signaling cascades in tissue repair, remodeling, fibrosis and regeneration, the members of the TGF-β superfamily have been considered attractive therapeutic targets for patients with myocardial infarction (190). Unfortunately, the pleiotropic and context-dependent actions of TGF-βs, the unique and complex biology of TGF-β activation, and the complexity of the signaling cascades activated by the members of the superfamily pose major challenges in therapeutic translation. Clearly, activation of TGF-β signaling affects phenotype and function of all cells involved in cardiac repair, exerting both protective and detrimental effects. Our current knowledge of the cell-specific actions of TGF-βs *in vivo* is not sufficient to design therapy. Moreover, the distinct actions of TGF-β-mediated Smad-dependent and non-Smad pathways add a layer of complexity that further complicates therapeutic translation. The pathophysiologic heterogeneity of myocardial infarction represents another major challenge. In the clinic, patients surviving acute myocardial infarction may exhibit very different responses, which are not always dependent on the extent of initial injury. Some patients tend to develop dilative remodeling that may be due to accentuated or unrestrained inflammatory activation. Other patients may have extensive fibrosis, associated with diastolic heart failure (191). Patient-specific TGF-β responses may be critical in determining the outcome in myocardial infarction. Thus, identification of patient subpopulations with overactive TGF-β signaling through the use of carefully validated biomarkers, or imaging studies, will be essential in order to design personalized treatment strategies. In any case, targeting the TGF-β system carries significant risks, because of its critical role in homeostasis and repair and its broad effects on many different cell types (192). For example, extensive evidence suggests that disruption of the TGF-β/Smad3 axis may promote aortic aneurysm formation and rupture (193–195). Thus, attempts for clinical translation of TGF-β/Smad inhibition strategies following infarction should focus on brief therapeutic interventions, and may need to exclude vulnerable patients.

## Author Contributions

All authors listed have made a substantial, direct and intellectual contribution to the work, and approved it for publication.

### Conflict of Interest

The authors declare that the research was conducted in the absence of any commercial or financial relationships that could be construed as a potential conflict of interest.
